# Prevention of Cholera

**Published:** 1885-12-20

**Authors:** 


PREVENTION OF CHOLERA.—The President alludes, in his message, to the International Conference which was held at Rome in May last to consider the means of arresting cholera and other epidemics, and states that "an expert delegate on behalf of the United States attended its sesions, and will submit a report."

